# Prevalence and Risk Factors of Poor Sleep Quality in Collegiate Athletes during COVID-19 Pandemic: A Cross-Sectional Study

**DOI:** 10.3390/ijerph19053098

**Published:** 2022-03-06

**Authors:** Marie-Anne Melone, Claire Tourny, Brian K. Gehlbach, Eli L. Schmidt, Matthieu Lalevée, Maxime L’Hermette

**Affiliations:** 1 Department of Pulmonary, Thoracic Oncology and Respiratory Intensive Care, Rouen University Hospital, Univ Rouen, F-76000 Rouen, France; 2 CETAPS EA3832, Research Center for Sports and Athletic Activities Transformations, University of Rouen Normandy, F-76821 Mont-Saint-Aignan, France; claire.tourny@univ-rouen.fr (C.T.); matthieu.lalevee@etu.univ-rouen.fr (M.L.); maxime.lhermette@univ-rouen.fr (M.L.); 3 Department of Internal Medicine, University of Iowa, Iowa City, IA 52240, USA; brian-gehlbach@uiowa.edu; 4 Department of Neurology, Carver College of Medicine, University of Iowa, Iowa City, IA 52240, USA; 5 Department of Orthopedics and Rehabilitation, Carver College of Medicine, University of Iowa, Iowa City, IA 52240, USA; eli-schmidt@uiowa.edu; 6 Department of Orthopedic Surgery, Rouen University Hospital, Univ Rouen, F-76000 Rouen, France

**Keywords:** student athletes, exercise, training, sleep disrupters, sleep deprivation, lifestyle habits, anxiety, mental health, circadian rhythms

## Abstract

The COVID-19 pandemic has changed our lifestyle, sleep and physical activity habits. This study evaluated the prevalence of poor sleep quality, its disrupters, and the impact of the pandemic in collegiate athletes. We performed a cross-sectional study of collegiate athletes (N = 339, median age: 20 (IQR,19–21) years old, 48.5% female, 47% individual sports) who received a web-based questionnaire in April 2021. This survey included subject characteristics, chronotype, sleep disrupters, the changes due to the pandemic and sleep quality (Pittsburg Sleep Quality Index [PSQI]). A multivariate linear regression was performed to assess the relationship between sleep quality, gender, chronotype, sleep disrupters and the changes to training volume or sleep. Results showed a disrupted sleep quality in 63.7%. One in five students had a total sleep time under 6.5 h per night. Poor sleep quality was significantly correlated with nocturnal concerns related to the pandemic, evening chronotype, female gender, third year of study, caffeine consumption and lack of sleep routine (all *p* < 0.05). To conclude, poor sleep quality is common in collegiate athletes. Sleep disrupters remain prevalent in the lifestyle habits of this population and may have been exacerbated by changes related to the COVID-19 pandemic. Sleep hygiene should become a major aspect of sports education during the return to post-covid normality.

## 1. Introduction

Sleep insufficiency is common in athletes. Some authors have reported a decrease in sleep duration and efficiency and an increase in sleep onset latency in athletes compared with non-athlete subjects [1,2]. Sleep disrupters are considered as any factor that negatively impacts any aspect of sleep. These disrupters can be those seen in the general population or can be more specifically related to athletics. Previous studies have shown that the amount of sleep is largely influenced by training programs; both early morning workouts [3] and evening training alter sleep duration [4]. Competitions [5], travel [6], and psychological pressure have also been shown to impair athletes’ sleep [7]. Additionally, individual sports athletes seem to obtain less sleep than athletes in team sports [8].

Sleep is a major component in cognition [9], learning and memory consolidation [10], well-being [11], cell growth and repair [12], glucose metabolism [13], secretion of leptin and ghrelin [14], and immune system function [15]. Therefore, longer sleep duration for athletes is important for recovery and energy conservation purposes [16]. Furthermore, sleep debt may lead to an increased risk of injuries [17], altered performance [18], prolonged recovery and loss of motivation [19].

Student athletes are also concerned regarding impaired quality and duration of sleep, especially because at their age a sleep duration of up to 8–10 h is recommended [20]. To date, 35–65% of collegiate athletes have been identified as poor sleepers by the Pittsburg Sleep Quality Index (PSQI) score [21,22,23]. Mah et al. reported in 628 Stanford University athletes that 42.4% experienced poor sleep quality and 40% had less than 7 h of sleep per night [24]. Despite this high prevalence, few studies have focused on identifying specific sleep disrupters in this population. 

Recently, a Japanese study of 906 collegiate athletes (mean age: 19, male: 70%) studied various sleep disrupters such as lifestyle habits (bedtime, wake-up time, smoking, drinking alcohol, meals, part-time jobs, and use of electronics after lights out), competition activities and psychological distress [25]. They reported risk factors for poor sleep quality (PSQI) related to late bedtime, early wake-up time, late night part-time jobs, use of smartphones after lights out, morning practices and psychological distress. The authors suggested that both lifestyle habits and psychological and training factors related to competitions can influence the sleep quality of student athletes. This study took place in 2018 outside of the context of the COVID-19 pandemic. Therefore, we were interested in investigating how these risk factors for poor sleep quality had changed during the COVID-19 pandemic.

The COVID-19 pandemic has been disrupting the world since December 2019. Social distancing and health recommendations to limit the spread of the virus have radically changed our lifestyle habits. Athletes have had to adapt to many new factors including new schedules, a decrease in volume of activity, a lack of social and team interactions, and the lack of competitions and motivation. Athletes may have had to deal with an even greater impact of these changes, given their goals related physical performance. 

A recent review of professional athletes focused on the impact of the pandemic on changes to physical activity, mental health and quality of life [26]. Fourteen studies and a total of 5434 subjects were included. They suggested that volume of training had decreased, stress, fatigue and depression had increased, and sleep quality had worsened during the pandemic. In this review, only two studies focused on sleep and included team sport athletes.

One reported sleep quality using only a Likert scale 1–10. Additionally, they reported the amount of training hours and days per week and the profile of mood state (POMS) early in the pandemic (April 2020) in 175 football players with mean age of 25 years old [27]. They found a decrease in volume training and sleep quality during the pandemic. Men had better sleep quality than women. Altered mood (POMS scores) had a significant effect on sleep quality and hours. It seemed that the amount of training days per week was positively correlated to sleep quality (r 0.35, *p* < 0.005).

The second study was also performed early in the pandemic (May 2020), in 565 athletes (mean age: 26.5, female: 57%, team-sport: 79%; elite: 42%) [28]. They used the Ultra-Short Munich Chronotype Questionnaire to estimate chronotype, the Single Daytime Sleepiness Item to assess daytime sleepiness, the Patient Health Questionnaire-4 to examine depression and anxiety, and asked questions to determine sleep pattern (time in bed, sleep onset latency, sleep need) and the amount of training hours and days per week. They demonstrated that, despite the increased sleep time, there were a delay in falling asleep and an increase in daytime sleepiness compared to before the pandemic among athletes. These changes were significantly associated with negative mental health outcomes. They highlighted the importance of considering individuals’ circadian rhythms, as the evening chronotype subjects had worse mental health outcome due to sleep timing changes. In their study, the authors did not evaluate sleep quality but instead reported sleep pattern and daytime sleepiness. Moreover, they did not report the impact of the type of sport (team vs. individual) or the level of practice (elite vs. non-elite).

Romdhani et al. investigated, in 3911 athletes (mean age: 25, female: 45%, team-sport: 63%, elite: 37%), COVID-19 mediated changes in training habits, circadian rhythms, sleep patterns, and eating behaviors to determine their link with sleep quality (PSQI) [29]. They found that the current period of lockdown led to a phase delay in the athlete’s circadian rhythms (longer sleep onset latency, later bedtime, and nocturnal eating) and reduced sleep quality. It seemed that elite and individual sports were more vulnerable to these disruptions, although decreased training volume wasn’t associated with a higher PSQI score. Despite the large sample size, this study had a heterogenous worldwide population but did not explore chronotype, or mental state.

Whether the reduction in athletes’ sleep quality during the COVID-19 pandemic is correlated with changes to training volume and competition conditions, circadian rhythms, sleep-wake schedules, lifestyle habits (meals, beverage consumption, screen time) or psychological distress is difficult to distinguish. Recent high-level studies lack concurrent evaluation of all these factors in the young athlete population. Knowing that athletes as well as youths are proven populations at risk for poor sleep quality and sleep deprivation, we deemed it relevant to study sleep quality in this population to clarify potential sleep disrupters related to training or mental state regarding the impact of the COVID-19 pandemic. To assess sleep quality, we assessed specific sleep disrupters related to training, including the PSQI as well as a specific validated athlete sleep screening questionnaire (ASSQ) [30]. We hypothesized there to be: (1) a high prevalence of poor sleep quality among collegiate athletes given their training and academic commitments, with significant changes in both of these areas during the COVID-19 pandemic as well as their chronobiologic predisposition to altered sleep quality; (2) poor sleep quality will be more pronounced in the event of a strong reduction in the volume of training, a high level of study and/or years of competing, when frequently worried at night (related to their sports and/or to the COVID-19 pandemic), and/or with inappropriate sleep hygiene (caffeine, sleep routine, use of naps, nighttime light exposure or training).

## 2. Methods

### 2.1. Study Design

This cross-sectional study was conducted to assess the sleep quality, and its disrupters, of collegiate athletes during the COVID-19 pandemic.

### 2.2. Procedure

The study was conducted between April and June 2021. The data were collected using an online questionnaire sent to each student’s electronic mailbox. At this time, students had not had in-person classes, competitions or in-person training sessions, as academic and training institutions had been closed since March 2020. The first and the most restrictive French confinement took place between 17 March and 3 May. After this first confinement, there were different partial confinements with lighter restrictions such as curfews and closures of public spaces that shaped the way of life of every French citizen during the completion of our study.

### 2.3. Ethics

The study was conducted according to the guidelines of the Declaration of Helsinki and approved by the Ethics Committee of the University of Rouen (protocol code E2021-69). Oral consent was given after the information was collected in this study and patients who did not consent to the protocol did not complete the questionnaire. The data collection was conducted in accordance with the MR004 reference methodology of the National Commission for Data Protection (Commission Nationale de l’Informatique et des Libertés) including patient information. Data were collected anonymously.

### 2.4. Participants

Participants in this study were students who registered for classes for fall 2020 at the University of Rouen, France. Students had to meet the following eligibility criteria: adult (>18 years old), registered in their 1st, 2nd, or 3rd year at the University of Rouen (Department of Sports and Exercise Science), elite and non-elite athletes, competing at any level, any type of sport and any duration of practice in their sport. Included students were those who answered the mandatory questions which allowed the returning of the questionnaire (some characteristics, severity of poor sleep and chronotype parts, Parts I and II Appendix A) and consented to the study (N = 352). Excluded students were those who sent back the questionnaire but for whom sleep disrupters section (Part III Appendix A) was not completed (N = 13).

### 2.5. Instruments

The questionnaire had three parts and consisted of 41 questions including 7 questions targeting population characteristics (1–7), 19 questions evaluated sleep quality (8–26), 1 question evaluated the chronotype (27), 4 questions targeted the impact of the COVID-19 pandemic on training volume and sleep quality (28–31), and the remaining questions used to detect sleep disrupters (32–41).

The questionnaire was written and distributed in the French language. However, the questionnaire is given in a full English version detailed in Appendix A.

#### 2.5.1. Population Characteristics

Questions 1 and 2 evaluated age and gender. From questions 3 to 7, we characterized the type of sport, the number of years of playing/competing, the level of practice, the number of hours of training per day and the year of study.

#### 2.5.2. Severity of Poor Sleep Quality and Chronotype

Questions 8 to 26 were composed of all the items needed to establish the PSQI [31] and the SDS [32]. The PSQI is composed of 7 sub-domains (subjective sleep quality, sleep latency, sleep duration, usual sleep efficiency, sleep disorders, medication use and daytime dysfunction) and participants must answer based on their habits for the previous month. Thus, this questionnaire gives a frequency for their sleep habits. The PSQI can range from 0 to 21. The higher the score, the worse the sleep quality. This was used to assess subjective sleep quality. We chose a PSQI score of 8 or more to discriminate moderate to severe disrupted sleep quality.

The ASSQ, an athlete specific questionnaire, is made up of five items used to assess the Sleep Difficulty Score (SDS). These five questions come from the PSQI to characterize (1) total sleep time, (2) satisfaction with sleep, (3) time taken to fall asleep, (4) trouble staying asleep and (5) use of sleep medications. However, the weighting is different from the PSQI. Therefore, the SDS can vary from 0 to 17, with 4 severity categories: None: 0–4, Mild: 5–7, Moderate: 8–10 or Severe: 11–17.

We assessed the chronotype using a single question from the Horne & Östberg Morningness–Eveningness Questionnaire (MEQ Question 19) as question 27 in our questionnaire [33]. This question has shown concordance with the full MEQ and been used in large cohorts [34,35,36,37]. On the other hand, we did not use the chronotype score from ASSQ since it was not validated against existing chronotype questionnaires or biological markers.

#### 2.5.3. Sleep Disrupters

For questions targeting the impact of the COVID-19 pandemic on training volume, the answers were divided according to the percentage of changes to the training volume (reduced by 30%, 60%, 90%, stable or increased training volume compared to before the pandemic). The impact of the COVID-19 pandemic on sleep was identified by a “yes or no” question and, if yes, dichotomized according to “deterioration” or “improvement” of sleep (Questions 28 to 31). The others ASSQ questions (Questions 32 to 36) were used to collect potential sleep disrupters, such as traveling to competitions, caffeine consumption, nighttime light exposure, and use of naps. Finally, we added sleep disrupters related to nocturnal concerns, whether about athletics or not related to practicing sports, along with sleep routine schedule and nighttime training (Questions 37 to 41).

### 2.6. Consensus for Data Treatment

From all questionnaires analyzed, only a few answers to questions in part I and III were missing. These missing data were excluded. Questions in part II were all answered since they were mandatory for returning the questionnaire. Therefore, we were able to obtain sleep quality by interpreting the PSQI score for all students analyzed. We obtained the gender in 338 (99.7%), the level of practice in 337 (99.4%), the number of hours of training per day in 335 (98.8%), the changes to training volume in 338 (99.7%) and sleep disturbances when travelling to competition in 307 (90.6%) of the total of 339 students.

### 2.7. Statistical Analysis 

Statistical analysis was obtained for all collected variables. Continuous measures were presented as means and standard deviations or medians and interquartile ranges. Categorical measures (lists of items and yes-no answers) are presented as counts and percentages. In univariate analyses, the 2-tailed Student test (*t*-test) for unpaired data, the Chi-square test of independency (two-tailed), and the 2-tailed Fisher’s exact test for categorical data were used to analyze the association between the variables of 3 different groups (PSQI (0–5), (6–11), (12–21)). A univariate association with *p*-value inferior to 0.2 was considered as the cut-off for including the variables in the multivariate analysis. A multivariate linear regression was performed to assess the relationship between PSQI and explanatory variables (gender, chronotype, year of study, years playing/competing in sport, changes to training volume and sleep, sports, and non-sports related concerns, travelling decreased performance, caffeine consumption, nighttime light exposure, use of naps, and sleep routine schedule). Data were verified for multicollinearity with the Belsley-Kuh-Welsch technique. The heteroscedasticity and normality of the variables were assessed by the White test and the Shapiro-Wilk test, respectively. Students with missing data were excluded from the analysis. A *p*-value < 0.05 was considered as statistically significant. The statistical analysis was carried out with Easy Med Stat (version 3.7; www.easymedstat.com, accessed on 1 July 2021). 

## 3. Results 

### 3.1. Population 

**Figure 1:**
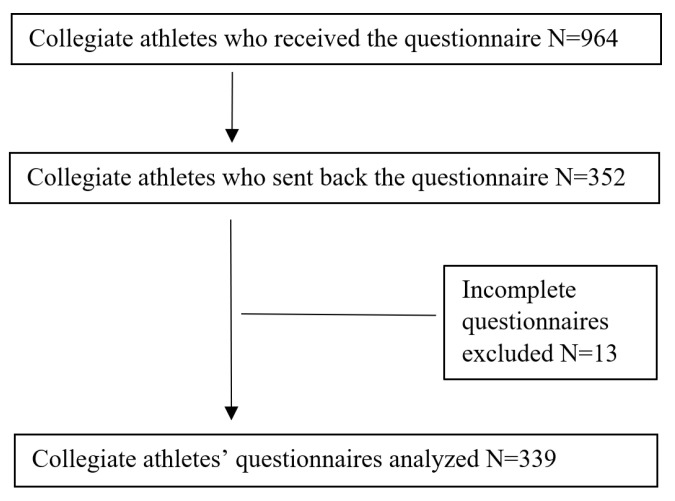
Flow diagram of the study.

We evaluated 339 sleep questionnaires among 964 students who received the questionnaire. Our response rate was 36.5% (Figure 1).

The median age was 20 (IQR,19–21) years old and 164/338 (48.5%) were female. The most commonly represented sports were football (15%), basketball (10%), handball (9.7%), swimming (7%) and volleyball (5.6%). Clinical and sport characteristics are presented in Table 1.

### 3.2. Sleep Characteristics

The prevalence of poor sleep quality (PSQI ≥ 8) was 216/339 (63.7%). One in five (67/339, 20%) students reported less than 6.5 h of sleep per night. The prevalence of moderate to severe alteration of sleep quality (SDS ≥ 8) was 126/339 (37.5%). The median PSQI was 9 (7–11) and the median SDS was 6 (4–9). The maximum PSQI score was 20 while the maximum SDS score was 17. Of this population, 65% had an intermediate chronotype (Table 2).

There was no significant difference in sleep quality or duration, use of sleep medication, or daytime enthusiasm between individual and team sports athletes. Athletes playing individual sports experienced more disruption in social activity due to poor sleep quality compared to athletes playing team sports (Table 3).

### 3.3. Sleep Disrupters

The most prevalent sleep disrupter was visual exposure to an electronic device within the hour before falling asleep (nighttime light exposure). Sports training after 7 p.m. (nighttime training) at least once a week was reported by 82.1% of students. Nocturnal concerns not related to sports was described “frequently” or “every night a week” by 36% of students. Frequent nocturnal concerns about sports were less reported (13%). Caffeine consumption was low with 90% of students reporting consumption of less than 3 units on average per day. Furthermore, 38% (118/307) and 21% (72/339) described sleep disturbances and decreased performance while travelling to competition, respectively, and 49% and 36% of students described a regular sleep routine at wake-up time and bedtime, respectively. 

Regarding the category of athletes, we found that athletes in individual sports complained more about breathing difficulties and temperature-related discomfort (Table S1); they had less night training, lower performance while traveling, and higher caffeine consumption than team sports athletes (Table S2). Athletes practicing at the national or international level had more night training and nocturnal concerns related to sports than athletes at the recreational level (Table S3).

### 3.4. Impact of COVID-19 

The impact of COVID-19 on training volume led to a 30–90% decrease in training volume in 81% of students. The impact of COVID-19 on sleep quality led to a deterioration of sleep in 159/339 (47%) students while 60/339 (18%) reported an improvement in their sleep quality (Table 1). Team sports athletes experienced a greater decrease in training volume due to the pandemic compared to individual sports athletes. National/international level athletes had more stable training volume than recreational level athletes. The impact of COVID-19 on sleep quality was not different by sport category (team vs. individual) and level of practice (national/international vs. recreational) (Tables S2 and S3).

From univariate analysis, changes to training volume were not significantly related to PSQI (*p* = 0.12). However, sleep deterioration experienced as a consequence of the COVID-19 pandemic was significantly related to greater values of PSQI (82.8%, 44.4%, 12% in group (12–21), (6–11), (0–5) respectively, *p* < 10^−4^, *p* < 10^−4^, *p* = 0.3 × 10^−4^) (Table S4). 

### 3.5. Predictors of Poor Sleep Quality in Multivariate Analysis

In multivariate analysis, evening chronotype (OR 1.36, 95% CI (0.67; 2.05), *p* < 0.001), female gender (OR 1.1, 95% CI (0.54; 1.65), *p* < 0.001), caffeine consumption (OR 1.09, 95% CI (0.18; 2.01), *p* = 0.02) and lack of sleep routine (OR 0.59, 95% CI (0.006; 1.18), *p* = 0.05) were significantly correlated with higher values of PSQI.

On the contrary, having few nocturnal concerns, feeling improved or stable sleep during COVID-19 pandemic (OR −2.54, 95% CI (−3.31; −1.76), *p* < 0.0001), and being in the 1st year of study (OR −0.91, 95% CI (−1.59; −0.24), *p* = 0.008) were associated with lower values of PSQI. Number of years of playing/competing, decreased training volume due to the pandemic, sport-specific nocturnal concerns, nighttime light exposure, and use of naps were not significantly associated with higher PSQI values (Table 4).

## 4. Discussion

Our study highlighted a high prevalence of moderate to severe poor sleep quality in collegiate athletes, significantly correlated with deterioration of sleep due to nocturnal concerns related to the COVID-19 pandemic, third year of study, caffeine consumption, lack of a sleep routine at bedtime, female gender, and evening chronotype. Thus, our first hypothesis regarding the prevalence of poor sleep quality among collegiate athletes was confirmed. However, our second hypothesis regarding the sleep disrupters was partially confirmed. It appeared that impaired sleep quality was more pronounced in students with high year of study and frequent nocturnal concerns related to the COVID-19 pandemic, but was unrelated to number of years of playing/competing, decreased training volume due to the pandemic, and concerns specifically related to their sport.

### 4.1. Prevalence of Poor Sleep Quality

Prevalence of poor sleep quality among collegiate athletes was high when expressed by the PSQI or SDS. This prevalence was similar to those reported in previous studies that found 40 to 60% of sports and non-sports students had impaired sleep quality [23,24,35]. For instance, Mah et al. reported that 42.4% students experienced poor sleep quality and 40% had less than 7 h of sleep per week [24]. A more recent study found an even higher rate of impaired sleep quality among student-athletes with 65% of their population identified as poor sleepers [22]. With our study, we confirmed that collegiate athletes are strongly affected by altered sleep quality.

### 4.2. Nocturnal Concerns

Our study reported that, in multivariate analysis, the deterioration of sleep due to nocturnal concerns related to the COVID-19 pandemic was the main factor correlated with poor sleep quality. Mental health disorders (depression, anxiety) have a high prevalence among university students [38]. Moreover, tension and stress accounted for 24% of the variance of the PSQI in students [23]. The COVID-19 pandemic has decreased the mental health and well-being of university students [39,40]. For instance, 50% of 115 first-year university students estimated their mental health to be worse than before the COVID-19 pandemic. Notably, males had fewer symptoms of anxiety and depression, compared with females [40]. The recent review mentioned above reported an increase in negative emotions in professional athletes during the COVID-19 pandemic [26]. Additionally, four validated psychological questionnaires were conducted in 169 professional and amateur athletes and showed that psychological states during lockdown were a predictor of the decrease in training and sleep quality [41]. In our study, nocturnal concerns related to the COVID-19 pandemic appeared to be the primary source leading to altered sleep quality. 

### 4.3. Year of Study

Athletes being in their third year of study was independently linked to higher values of PSQI. Regarding this finding, existing literature provides controversial hypotheses. Some studies have found that students in the early years of college had a higher prevalence of poor sleep quality compared to those in later years, arguing that younger students might be more susceptible due to lack of experience in managing the rigorous new academic schedule [24,42]. However, it is likely that older students were exposed to sleep disrupters related to their sport and/or academic conditions for longer than the rest of the younger cohort [43]. In our cohort, students in higher years of study had poorer sleep quality.

### 4.4. Training Volume

We hypothesized a negative impact on sleep quality due to the changes seen in the training volume. Physical activity is known to be associated with better mental health in the general population [44]. Therefore, we thought that the decrease in physical activity in the athlete’s population could have been a major source of decreased sleep quality. In our study, although the COVID-19 pandemic caused a decrease in training volume in 81% of students, this decrease was not significantly correlated with higher PSQI values. Other recent studies have proven the significant decrease in the number of workouts per week during the lockdown compared to before the pandemic [26]. However, the COVID-19-induced phase delay with shift to later sleeping hours on working days as well as weekend days, elegantly shown by Facer-Childs et al. and Romdhani et al., seemed to play the major role in the deterioration of sleep quality [28,29]. Athletes with an evening chronotype, compared to a morning chronotype, were more affected by these changes. 

### 4.5. Chronotype

In our study we found that evening chronotype influenced sleep quality during the pandemic more than the change to training volume. The relationship between poor sleep quality and evening chronotype is in line with established knowledge about delayed phase syndrome in adolescents and young adults and its adverse consequences on sleep duration, physical and mental health [45]. Moreover, this emphasizes the importance of considering circadian phenotypes in collegiate athletes because of their relationship with sleep and performance [46,47]. 

### 4.6. Gender

Recent literature has found female gender to be associated with poor sleep quality and anxiety disorders during the pandemic [48,49]. Notably, in a study among 13,989 Italians, one of the main predictors of poor sleep quality and insomnia was female gender [50]. However, women may be more willing to express their feelings and psychological symptoms compared with men. In a study assessing the prevalence of sleep deprivation among college athletes, most male and female college athletes received sleep levels below recommended age-based sleep levels as measured by actigraphy. However, male athletes significantly overestimated their total sleep time when self-reported in the PSQI questionnaire [21]. 

### 4.7. Sleep Hygiene

Overall, although the origins of the degradation of sleep quality induced during the COVID-19 pandemic are numerous, complex and entangled, as reported in our own and other recent studies, sleep hygiene has been greatly perturbed. 

In this cohort of young athletes with poor sleep quality, not only may their type of sport and training influence their sleep quality, but other basic principles of sleep hygiene were not followed (caffeine consumption and lack of a sleep routine). Athletes would benefit from implementing these basic principles of sleep hygiene to improve the quality of sleep. Sleep hygiene protocols contain basic and fundamental rules that are easy to follow in order to optimize the restorative effect of sleep and reinforce its benefits on metabolic, immune, and cognitive functions that can improve the performance of athletes. Spreading sleep hygiene protocols is an easy, quick, and effective way to address poor sleep quality. Therefore, it seems relevant to insist on the dissemination of these rules of sleep hygiene among the sports population:

- Respect 7 to 9 h of sleep at night, even 8 to 10 for teenagers
- Respect the sleep routine: go to bed and wake up at the same time every day (±30 min, even on weekend days)
- Respect your chronotype to plan an adequate bedtime, wake-up time and training
- Practice relaxation techniques before bedtime
- Go to bed when sleepy, do not stay awake for more than 30 min
- Avoid caffeine within 6 h of bedtime
- Respect adequate exposure to natural light in the morning
- Do not look at blue-light electronic devices before bedtime
- Avoid heavy exercises at the end of the evening
- Avoid inappropriate naps (prefer naps about 10 min after lunchtime)
- Use a relaxing bedtime routine and an appropriate sleep environment (e.g., cool, dark, quiet)

### 4.8. Limits

Our study has certain limitations. First, our prevalence is based on the population that was willing to answer the questionnaire and might be biased. However, our response rate is coherent with the literature that reports a lower response rate when using online versus in-person surveys [51,52]. Second, we did not have an a priori power calculation due to the lack of bibliographic evidence regarding this cohort in the pandemic context at the time we started it. Therefore, we tried to obtain the largest possible cohort. Another limitation is the cross-sectional design which makes it difficult to determine causality between risk factors and sleep quality. 

Additionally, it could be hypothesized that, during the lockdown, since athletes had their training volume decreased, they would sleep more to recover from chronic sleep debt. We reported sleep duration (total sleep time, Table 2) during the pandemic, but we did not report the sleep schedules pre-pandemic. It would be possible that sleep duration before the pandemic was shorter than that which we found. Other recent studies found an increase in sleep duration of 13 to 36 min during lockdown [53]. However along with increased sleep duration they found decreased sleep quality, suggesting that longer sleep duration does not lead necessarily to better sleep quality and other contributors may alter sleep quality, such as mental health. It also may be a discrepancy between self-reported sleep duration overestimated compared with objective polysomnography sleep duration. Indeed, sleep times were collected based on a subjective analysis without this being corroborated by an objective measure (actigraphy or polysomnography). However, our study aimed to assess the sleep quality and PSQI has shown high reliability and good validity in patients with insomnia [54]. Large cohort studies tend to make these objective measurements impossible due to the cost and labor demands. 

The subjective assessment of the change of sleep quality before/after the pandemic required a reminder one year apart and may have contributed to a recall bias. We did not report the sleep schedules pre-pandemic as other studies did. Rather, we asked for a subjective assessment of “the change of sleep quality due to the COVID-19 pandemic” (Question 31). Therefore, we did not compare PSQI score before/after pandemic.

Our objective was to assess sleep disrupters specifically related to the collegiate athlete population. To do so, we used a 41 item questionnaire, composed from two validated questionnaires. Indeed, PSQI and ASSQ are clinically validated in the general population and the athlete’s population, respectively. However, to assess sleep disrupters specifically related to the collegiate athlete population the significant changes in lifestyle habits due to the COVID-19 pandemic may have masked the relationship between these disrupters (i.e., training after 7 p.m., travelling and competition) and poor sleep quality that may exist outside the context of the pandemic. Another important factor could be that the COVID-19 infection itself, due to its symptoms such as fatigue and shortness of breath, could have led directly to worsened sleep quality, but we did not record the percentage of patients having had COVID-19 in our cohort. 

## 5. Conclusions

This study reported a high prevalence of poor sleep quality among collegiate athletes. Nocturnal concerns related to the COVID-19 pandemic, female gender, evening chronotype, third year of study, caffeine consumption and lack of sleep routine were independently associated with poor sleep quality.

Sleep quality is the cornerstone of athlete performance, recovery, and well-being. The COVID-19 pandemic has led to changes in lifestyle, sleep patterns, and training, as well as increased psychological distress, all combining to reduce the quality and quantity of sleep in collegiate athletes. Sleep disrupters remain in the lifestyle habits of many young athletes and may have been exacerbated by changes related to the COVID-19 pandemic. Therefore, sleep hygiene should be improved and become a major aspect of sports education during the return to post-covid normality.

## Figures and Tables

**Table 1:** Characteristics of collegiate athletes.

Variables	Modality	Values
		median (IQR) or n (%)
Total		339
Age (Years)		20 (19–21)
Gender	Female	164 (48,5)
Type of sport	Individual	161 (47)
	Team	178 (53)
Years playing/competing	<5	72 (21)
	5–10	107 (32)
	11–15	114 (34)
	>15	46 (14)
Level of practice	Departmental	114 (34)
	Regional	154 (46)
	National	64 (19)
	International	5 (1)
Hours/day of training	<2	185 (55)
	2–3	102 (30)
	4–5	33 (10)
	>5	15 (4)
Year of school study	1st	96 (28)
	2nd	104 (31)
	3rd	139 (41)
Changes to training volume	No impact	35 (10)
	Decrease by 30%	83 (24)
	Decrease by 60%	124 (37)
	Decrease by 90%	67 (20)
	Increase	29 (9)
Changes to sleep	No impact	120 (35)
	Deterioration	159 (47)
	Improvement	60 (18)

**Table 2:** Sleep characteristics of collegiate athletes.

Variables		Values	95% CI
		median (IQR) or n (%)	
Total		339	
Sleep quality	PSQI	9 (7–11)	[8.4–9.1]
Sleep quantity	TST	8 (7–8.3)	[7.4–7.7]
	TIB	9 (8–10)	[8.6–8.9]
	Efficacy	88 (80–95)	[84.7–87.2]
Chronotype	Evening	74 (22)	
	Intermediate	221 (65)	
	Morning	44 (13)	

PSQI, Pittsburg sleep quality index; TST, Total sleep time; TIB, Time intra-bed.

**Table 3:** Sleep characteristics based on sports category.

Variables	Modality or Frequency *	Values Median (IQR) or n (%)		*p*-Value
Sport		Individual	Team	
Total		161	178	
PSQI		9 (7–11)	9 (7–10)	0.6 ^a^
SDS		6 (4–9)	6 (4–9)	1 ^a^
Subjective sleep quality	Very good	22 (14)	19 (11)	0.9 ^b^
	Fairly good	74 (46)	87 (49)	
	Fairly bad	55 (34)	61 (34)	
	Very bad	10 (6)	11 (6)	
Sleep latency (min)	0–15	50 (31)	60 (34)	0.2 ^b^
	16–30	50 (31)	40 (22)	
	31–60	40 (25)	42 (24)	
	>60	21 (13)	36 (20)	
Difficulty falling asleep	No	19 (12)	27 (15)	0.2 ^b^
	<1	60 (37)	46 (26)	
	1–2	35 (22)	42 (24)	
	≥3	47 (29)	63 (35)	
Use of sleep medication	None	142 (88)	167 (94)	0.06 ^c^
	<1	10 (6)	2 (1)	
	1–2	2 (1)	1 (1)	
	≥3	7 (4)	8 (5)	
Social activity disturbances	None	66 (41)	104 (58)	0.01 ^c^
	<1	63 (40)	52 (29)	
	1–2	26 (16)	17 (10)	
	≥3	6 (4)	5 (3)	
Lack of enthusiasm	not a problem	49 (30)	59 (33)	0.9 ^b^
	only a slight	47 (29)	47 (26)	
	somewhat	44 (27)	52 (29)	
	a big problem	21 (13)	20 (11)	

* Number of times per week in the past month, ^a.^ 2-tailed Student test (*t*-test) for unpaired data, ^b.^ Chi-square test of independency (two-tailed), ^c.^ 2-tailed Fisher’s exact test for categorical data. PSQI, Pittsburg sleep quality index; SDS, Sleep difficulty score.

**Table 4:** Multivariate analysis of PSQI as dependent variable.

Variable	Modality	Odds Ratio, 95% CI	*p*-Value
Intercept		9.68 [8.77; 10.6]	<0.0001
Gender	Female	1.1 [0.54; 1.65]	<0.001
Chronotype	Intermediate (reference)		
	Evening	1.36 [0.67; 2.05]	<0.001
	Morning	0.25 [−0.58; 1.08]	0.6
Years playing/competing in sport	>10 years	0.07 [−0.48; 0.62]	0.8
Year of school study	3rd year (reference)		
	1st year	−0.91 [−1.59; −0.24]	0.008
	2nd year	−0.63 [−1.29; 0.03]	0.06
COVID-19 impact on training volume	Increase or stable volume	−0.43 [−1.15; 0.3]	0.3
COVID-19 impact on sleep	Deterioration (reference)		
	Improvement	−2.54 [−3.31; −1.76]	<0.0001
	No impact	−2.48 [−3.13; −1.84]	<0.0001
Traveling decreased performance	Yes	0.48 [−0.19; 1.14]	0.2
Caffeine consumption	>2 units/day	1.09 [0.18; 2.01]	0.02
Nighttime light exposure	Every night	0.18 [−0.68; 1.04]	0.7
Sleep concerns related to sports	Sometimes-Frequently-Always	0.5 [−0.12; 1.11]	0.1
Sleep concerns not related to sports	Never-Rarely	−1.43 [−2.06; −0.80]	<0.0001
Number of naps per week	At least 1 per week	−0.064 [−0.61; 0.48]	0.8
Sleep routine schedule at bedtime	Never-Rarely	0.59 [0.006; 1.18]	0.05

## Data Availability

The data presented in this study are available on request from the corresponding author. The data are not publicly available.

## Supplementary Materials

The following supporting information can be downloaded at: https://www.mdpi.com/article/10.3390/ijerph19053098/s1, Table S1: Sleep disrupters from PSQI answers based on sport’s category. Table S2: Other sleep disrupters based on sport’s category. Table S3: Other sleep disrupters based on level of practice. Table S4: Univariate analyses of sleep disrupters between three different groups according to PSQI.

## Appendix A

Sleep quality and disrupters screening questionnaire among collegiate athletes during the COVID-19 pandemic [31,32].
I **First part: population characteristics**
1. Are you?
A woman
A man
2. What is your date of birth?
…………………………
3. What type of sport do you practice?
…………………………
4. How many years have you been playing/competing in your sport?
Less than 5 years
5 to 10 years
11 to 15 years
More than 15 years
5. What level of practice are you in?
Departmental
Regional
National
International
6. How many hours of training per day do you do?
Less than 2 h
2 to 3 h
4 to 5 h
More than 5 h
7. What year of school study are you in?
1 st year
2 nd year
3 rd year
II **Second part: Severity of poor sleep quality and chronotype**
8–26 Questions as expressed by Buysse et al. [31] and Samuels et al. [32] to score the Pittsburg Sleep Quality Index (PSQI) and the Sleep Difficulty Score (SDS).
27. Do you consider yourself to be a morning type person or an evening type person?
Definitely a morning type
More a morning type than an evening type
More an evening type than a morning type
Definitely an evening type
III **Third part: Sleep disrupters**
28. Do you think your training volume has been impacted by the COVID-19 pandemic?
Yes
No
29. If yes, how has your training volume changed due to the COVID-19 pandemic?
Decreased by 30%
Decreased by 60%
Decreased by 90%
Increased
30. Do you think your sleep quality has been impacted by the COVID-19 pandemic?
Yes
No
31. If yes, how has your sleep quality changed due to the COVID-19 pandemic?
Deterioration
Improvement
32. When you are travelling for your sport, do you experience sleep disturbance?
Yes
No
33. When you are travelling for your sport, do you experience a decreased performance?
Yes
No
34. During the past month, how much caffeine do you drink per day?
* (1 unit = for coffee or tea 150–250 mL, for* caffeinated *drink 350 mL)*
Less 1 unit
1 to 2 units
3 units
More than 4 units
35. During the past month, how often do you use an electronic device within the hour preceding sleep? *(night per week)*
Never
1 to 3 nights
4 to 6 nights
Every night
36. During the past month, how many naps per week do you take?
None
Once or twice
Three or four
Five to seven
37. Before pandemic, how often did you train after 7:00 PM per week?
Not at all
1 to 3 times
4 to 6 times
Every night
38. During the past month, how often do you have sleep concerns related to sport?
Never
Rarely
Sometimes
Frequently
Always
39. During the past month, how often do you have sleep concerns not related to sport?
Never
Rarely
Sometimes
Frequently
Always
40. During the past month, do you get up every morning at the same time?
(*less than 1 h* variation)
Never
Rarely
Sometimes
Frequently
Always
41. During the past month, do you go to bed every night at the same time?
(*less than 1 h* variation)
Never
Rarely
Sometimes
Frequently
Always
