# Supplementary material for: Prevalence and Risk Factors of Poor Sleep Quality in Collegiate Athletes during COVID-19 Pandemic: A Cross-Sectional Study
Source: Int J Environ Res Public Health. 2022 Mar 6;19(5):3098. doi: 10.3390/ijerph19053098 (PMC8910097; doi:10.3390/ijerph19053098)
Supplement: Supplementary file 1 [file ijerph-19-03098-s001.zip › Appendix 1 2.18.22.pdf]

Appendix 1. Sleep quality and disrupters screening questionnaire among collegiate athletes during the COVID-19 pandemic:

I. First part: population characteristics

1. Are you?
   A woman
   A man

2. What is your date of birth?
   …………………………

3. What type of sport do you practice?
   …………………………

4. How many years have you been playing/competing in your sport?
   Less than 5 years
   5 to 10 years
   11 to 15 years
   More than 15 years

5. What level of practice are you in?
   Departmental
   Regional
   National
   International

6. How many hours of training per day do you do?
   Less than 2 h
   2 to 3 h
   4 to 5 h
   More than 5 h

7. What year of school study are you in?
   1 st year
   2 nd year
   3 rd year

II. Second part: Severity of poor sleep quality and chronotype

8-26 Questions as expressed by Buysse et al.(1) and Samuels et al.(2) to score the Pittsburg Sleep Quality Index (PSQI) and the Sleep Difficulty Score (SDS).

27. Do you consider yourself to be a morning type person or an evening type person?
    Definitely a morning type
    More a morning type than an evening type
    More an evening type than a morning type
    Definitely an evening type

III. Third part: Sleep disrupters

28. Do you think your training volume has been impacted by the COVID-19 pandemic?
    - Yes
    - No

29. If yes, how has your training volume changed due to the COVID-19 pandemic?
    - Decreased by 30 %
    - Decreased by 60%
    - Decreased by 90 %
    - Increased

30. Do you think your sleep quality has been impacted by the COVID-19 pandemic?
    - Yes
    - No

31. If yes, how has your sleep quality changed due to the COVID-19 pandemic?
    - Deterioration
    - Improvement

32. When you are travelling for your sport, do you experience sleep disturbance?
    - Yes
    - No

33. When you are travelling for your sport, do you experience a decreased performance?
    - Yes
    - No

34. During the past month, how much caffeine do you drink per day?
(1 unit = for coffee or tea 150-250 mL, for caffeinated drink 350 mL)
    - Less 1 unit
    - 1 to 2 units
    - 3 units
    - More than 4 units

35. During the past month, how often do you use an electronic device within the hour preceding sleep? *(night per week)*
    - Never
    - 1 to 3 nights
    - 4 to 6 nights
    - Every night

36. During the past month, how many naps per week do you take?
    - None
    - Once or twice

Three or four
Five to seven

37. Before pandemic, how often did you train after 7:00 PM per week?
Not at all
1 to 3 times
4 to 6 times
Every night

38. During the past month, how often do you have sleep concerns related to sport?
Never
Rarely
Sometimes
Frequently
Always

39. During the past month, how often do you have sleep concerns not related to sport?
Never
Rarely
Sometimes
Frequently
Always

40. During the past month, do you get up every morning at the same time?
(less than 1 hour variation)
Never
Rarely
Sometimes
Frequently
Always

41. During the past month, do you go to bed every night at the same time?
(less than 1 hour variation)
Never
Rarely
Sometimes
Frequently
Always

References:
1. Buysse DJ, Reynolds CF, 3rd, Monk TH, Berman SR, Kupfer DJ. The Pittsburgh Sleep Quality Index: a new instrument for psychiatric practice and research. Psychiatry Res. 1989;28(2):193-213.
2. Samuels C, James L, Lawson D, Meeuwisse W. The Athlete Sleep Screening Questionnaire: a new tool for assessing and managing sleep in elite athletes. Br J Sports Med. 2016;50(7):418-22.
