# Supplementary material for: Prevalence and Risk Factors of Poor Sleep Quality in Collegiate Athletes during COVID-19 Pandemic: A Cross-Sectional Study
Source: Int J Environ Res Public Health. 2022 Mar 6;19(5):3098. doi: 10.3390/ijerph19053098 (PMC8910097; doi:10.3390/ijerph19053098)
Supplement: Supplementary file 1 [file ijerph-19-03098-s001.zip › Supplementary material 2.22.22 .pdf]

**Table S1.** Sleep disrupters from PSQI answers based on sport's category.

| **Variables** | **Frequency*** | **Values** | | ***P-value*** |
|---|---|---|---|---|
| **Sport** | | Individual | Team | |
| **Total** | | 161 | 178 | |
| **Early wake up** | None | 19 (12) | 31 (17) | *0.1* [b] |
| | < 1 | 50 (31) | 43 (24) | |
| | 1 - 2 | 37 (23) | 54 (31) | |
| | ≥ 3 | 55 (34) | 50 (28) | |
| **Use the bathroom** | None | 64 (40) | 91 (51) | *0.1* [b] |
| | < 1 | 44 (27) | 33 (19) | |
| | 1 - 2 | 28 (17) | 27 (15) | |
| | ≥ 3 | 25 (16) | 27 (15) | |
| **Cannot breathe** | None | 106 (66) | 144 (81) | ***0.008*** [c] |
| | < 1 | 33 (21) | 19 (11) | |
| | 1 - 2 | 15 (9) | 13 (7) | |
| | ≥ 3 | 7 (4) | 2 (1) | |
| **Cough or snore** | None | 99 (61) | 98 (55) | *0.08* [b] |
| | < 1 | 35 (22) | 53 (30) | |
| | 1 - 2 | 13 (8) | 20 (11) | |
| | ≥ 3 | 14 (9) | 7 (4) | |
| **Too cold** | None | 63 (39) | 82 (46) | *0.2* [b] |
| | < 1 | 44 (27) | 54 (30) | |
| | 1 - 2 | 41 (26) | 30 (17) | |
| | ≥ 3 | 13 (8) | 12 (7) | |
| **Too hot** | None | 46 (29) | 64 (36) | ***0.01*** [b] |
| | < 1 | 49 (30) | 60 (34) | |
| | 1 - 2 | 54 (34) | 33 (18) | |
| | ≥ 3 | 12 (7) | 21 (12) | |
| **Bad dreams** | None | 48 (30) | 48 (27) | *0.2* [b] |
| | < 1 | 53 (33) | 73 (41) | |
| | 1 - 2 | 44 (27) | 48 (27) | |
| | ≥ 3 | 16 (10) | 9 (5) | |
| **Pain** | None | 60 (37) | 77 (43) | *0.09* [b] |
| | < 1 | 53 (33) | 49 (28) | |
| | 1 - 2 | 28 (17) | 41 (23) | |
| | ≥ 3 | 20 (13) | 11 (6) | |

***** *Number of times per week in the past month,*

*Statistical analysis: median [IQR], number (percentage), a. 2-tailed Student test (t-Test) for unpaired data, b. Chi-square test of independency (two-tailed), c. 2-tailed Fisher's exact test for categorical data.*

**Table S2.** Other sleep disrupters based on sport's category.

| **Variables** | **Modality** | **Values** | | ***P-value*** |
|---|---|---|---|---|
| Sport | | Individual | Team | |
| Total | | 161 | 178 | |
| Impact of COVID-19 on training volume | No impact | 21 (13) | 14 (8) | ***0.002*** [a] |
| | Decrease by 30 % | 43 (27) | 40 (22) | |
| | Decrease by 60% | 53 (33) | 71 (40) | |
| | Decrease by 90 % | 22 (14) | 45 (25) | |
| | Increase | 21 (13) | 8 (5) | |
| on sleep | No impact | 61 (38) | 59 (33) | *0.6* [a] |
| | Deterioration | 71 (44) | 88 (50) | |
| | Improvement | 29 (18) | 31 (17) | |
| Caffeine consumption (Unit / Day) | < 1 | 95 (59) | 136 (76) | ***0.04*** [b] |
| | 1 - 2 | 47 (29) | 31 (18) | |
| | ≥ 3 | 19 (12) | 8 (5) | |
| Training after 7:00 PM (/ Week) | None | 42 (26) | 18 (10) | ***0.0007*** [b] |
| | 1 - 3 | 91 (57) | 120 (67) | |
| | 4 - 6 | 26 (16) | 33 (19) | |
| | 7 | 2 (1) | 7 (4) | |
| Sleep disturbances when travelling to competition | Yes | 60 (43) | 58 (35) | *0.2* [b] |
| Decreased performance when travelling | Yes | 44 (27) | 28 (16) | ***0.01*** [b] |
| Electronic device before falling asleep (/ Week) | None | 0 (0) | 1 (1) | *0.2* [b] |
| | 1 - 3 | 8 (5) | 4 (2) | |
| | 4 - 6 | 15 (9) | 10 (5) | |
| | 7 | 138 (86) | 163 (92) | |
| Sleep concerns related to sports | Never | 50 (31) | 60 (34) | *0.07* [b] |
| | Rarely | 46 (29) | 51 (29) | |
| | Sometimes | 43 (27) | 46 (25) | |
| | Frequently | 15 (9) | 21 (12) | |
| | Always | 7 (4) | 0 (0) | |
| Sleep concerns not related to sports | Never | 28 (17) | 37 (21) | *0.2* [a] |
| | Rarely | 22 (14) | 37 (21) | |
| | Sometimes | 47 (29) | 51 (28) | |
| | Frequently | 45 (28) | 41 (23) | |
| | Always | 19 (12) | 12 (7) | |
| Naps (/ Week) | None | 88 (55) | 99 (55) | *0.9* [a] |
| | 1 - 2 | 53 (33) | 59 (33) | |
| | 3 - 4 | 14 (8) | 12 (7) | |
| | 5 - 7 | 6 (4) | 8 (5) | |
| Do you get up every morning at the same time? | Never | 9 (5) | 10 (6) | *0.9* [a] |
| | Rarely | 26 (16) | 36 (20) | |
| | Sometimes | 46 (29) | 48 (27) | |
| | Frequently | 74 (46) | 78 (44) | |
| | Always | 6 (4) | 6 (3) | |
| Do you go to bed every night at the same time? | Never | 12 (7) | 25 (14) | *0.05* [b] |
| | Rarely | 37 (23) | 37 (21) | |
| | Sometimes | 44 (27) | 62 (35) | |
| | Frequently | 67 (42) | 52 (29) | |
| | Always | 1 (1) | 2 (1) | |

*Statistical analysis: median [IQR], number (percentage), a. 2-tailed Student test (t-Test) for unpaired data, b. Chi-square test of independency (two-tailed), c. 2-tailed Fisher's exact test for categorical data.*

**Table S3. Other sleep disrupters based on level of practice.**

| Variables | Modality | Values | | *P-value* |
|---|---|---|---|---|
| Practice | | Recreational | National/International | |
| Total | | 268 | 69 | |
| PSQI | | 9 [7-11] | 8 [7-9] | *0.09* [a] |
| SDS | | 6.5 [4-6] | 5 [3-6] | *0.05* [a] |
| Impact of COVID-19 on training volume | No impact | 23 (9) | 11 (16) | ***0.005*** [b] |
| | Decrease by 30 % | 73 (27) | 10 (15) | |
| | Decrease by 60% | 101 (38) | 23 (33) | |
| | Decrease by 90 % | 44 (16) | 22 (32) | |
| | Increase | 26 (10) | 3 (4) | |
| on sleep | No impact | 89 (33) | 29 (42) | *0.1* [c] |
| | Deterioration | 134 (50) | 25 (36) | |
| | Improvement | 45 (17) | 15 (22) | |
| Caffeine consumption (Unit / Day) | < 1 | 177 (66) | 49 (71) | *0.6* [b] |
| | 1 - 2 | 65 (24) | 13 (19) | |
| | ≥ 3 | 26 (10) | 7 (10) | |
| Training after 7:00 PM (/ Week) | None | 51 (19) | 8 (11) | ***0.0001*** [b] |
| | 1 - 3 | 177 (66) | 33 (48) | |
| | 4 - 6 | 37 (14) | 22 (32) | |
| | 7 | 3 (1) | 6 (9) | |
| Sleep disturbances when travelling to competition | Yes | 92 (39) | 26 (38) | *1* [b] |
| Decreased performance when travelling | Yes | 61 (23) | 10 (15) | *0.2* [b] |
| Electronic device before falling asleep (/ Week) | None | 1 (0.4) | 0 (0) | *0.4* [b] |
| | 1 - 3 | 10 (4) | 2 (3) | |
| | 4 - 6 | 23 (8.6) | 2 (3) | |
| | 7 | 234 (87) | 65 (94) | |
| Sleep concerns related to sports | Never | 86 (32) | 24 (35) | ***0.01*** [b] |
| | Rarely | 85 (32) | 10 (14) | |
| | Sometimes | 69 (26) | 20 (29) | |
| | Frequently | 23 (8) | 13 (19) | |
| | Always | 5 (2) | 2 (3) | |
| Sleep concerns not related to sports | Never | 50 (19) | 15 (22) | *0.2* [b] |
| | Rarely | 49 (18) | 10 (15) | |
| | Sometimes | 70 (26) | 27 (39) | |
| | Frequently | 71 (27) | 14 (20) | |
| | Always | 28 (10) | 3 (4) | |
| Naps (/ Week) | None | 147 (55) | 39 (57) | *0.5* [b] |
| | 1 - 2 | 91 (34) | 20 (29) | |
| | 3 - 4 | 21 (8) | 5 (7) | |
| | 5 - 7 | 9 (3) | 5 (7) | |
| Do you get up every morning at the same time? | Never | 13 (5) | 6 (9) | *0.7* [b] |
| | Rarely | 50 (19) | 11 (16) | |
| | Sometimes | 74 (28) | 19 (27) | |
| | Frequently | 122 (45) | 30 (44) | |
| | Always | 9 (3) | 3 (4) | |
| Do you go to bed every night at the same time? | Never | 28 (10) | 9 (13) | *0.7* [b] |
| | Rarely | 57 (21) | 16 (23) | |
| | Sometimes | 87 (33) | 19 (28) | |
| | Frequently | 94 (35) | 24 (35) | |
| | Always | 2 (1) | 1 (1) | |

PSQI, Pittsburg sleep quality index; SDS, Sleep difficulty score.

<u>Statistical analysis</u>: *median [IQR], number (percentage), a. 2-tailed Student test (t-Test) for unpaired data, b. Chi-square test of independency (two-tailed), c. 2-tailed Fisher's exact test for categorical data.*

**Table S4.** Univariate analyses of sleep disrupters between three different groups according to PSQI.

| **Variables** | **Modality** | **PSQI [0- 5] (Group1) N = 50** | **PSQI [6- 11] (Group2) N = 225** | **PSQI [12-21] (Group3) N = 64** | ***p Group 1*2*** | ***p Group 2*3*** | ***p Group 1*3*** |
|---|---|---|---|---|---|---|---|
| Age | | 20 [19 - 20] | 20 [19 - 21] | 20 [19 - 21] | *0.5* [a] | *0.8* [a] | *0.5* [a] |
| Gender | Male | 37 (74) | 110 (49) | 27 (42) | *0.002* [b] | *0.32* [b] | *0.001* [b] |
| | Female | 13 (26) | 114 (51) | 37 (58) | | | |
| Chronotype | Evening | 10 (20) | 41 (18) | 23 (36) | *0.9* [b] | *0.01* [b] | *0.2* [b] |
| | Intermediate | 34 (68) | 153 (68) | 34 (53) | | | |
| | Morning | 6 (12) | 31 (14) | 7 (11) | | | |
| Impact of COVID-19 | | | | | | | |
| on training volume | No impact | 8 (16) | 24 (11) | 3 (5) | *0.4* [b] | *0.3* [b] | *0.1* [b] |
| | Decrease by 30 % | 12 (24) | 57 (25) | 14 (22) | | | |
| | Decrease by 60% | 14 (27) | 79 (35) | 31 (48) | | | |
| | Decrease by 90 % | 9 (18) | 47 (21) | 11 (17) | | | |
| | Increase | 6 (12) | 18 (8) | 5 (8) | | | |
| on sleep | No impact | 33 (66) | 79 (35) | 8 (12) | *$0.3*10^{-4}$* [c] | *< $10^{-4}$* [b] | *< $10^{-4}$* [b] |
| | Deterioration | 6 (12) | 100 (45) | 53 (83) | | | |
| | Improvement | 11 (22) | 46 (20) | 3 (5) | | | |
| Sleep Difficulty Score, SDS | | 3 [1 - 4] | 5 [4 - 7] | 10 [8 - 11] | *< $10^{-4}$* [a] | *< $10^{-4}$* [a] | *< $10^{-4}$* [a] |
| Caffeine consumption (Unit / Day) | < 1 | 35 (70) | 156 (69) | 37 (58) | *1* [b] | *0.04* [b] | *0.1* [b] |
| | 1 - 2 | 13 (26) | 50 (22) | 15 (23) | | | |
| | ≥ 3 | 2 (4) | 19 (9) | 12 (19) | | | |
| Training after 7:00 PM (/ Week) | None | 12 (24) | 39 (17) | 9 (14) | *0.8* [b] | *0.7* [b] | *0.5* [b] |
| | 1 - 3 | 30 (60) | 142 (63) | 39 (61) | | | |
| | 4 - 6 | 7 (14) | 38 (17) | 14 (22) | | | |
| | 7 | 1 (2) | 6 (3) | 2 (3) | | | |
| Sleep disturbances when travelling to competition | Yes | 7 (16) | 80 (39) | 31 (53) | *0.003* [b] | *0.07* [b] | *< $10^{-4}$* [b] |
| | No | 38 (84) | 124 (61) | 27 (47) | | | |
| Decreased performance when travelling to competition | Yes | 6 (12) | 50 (22) | 16 (25) | *0.12* [b] | *0.62* [b] | *0.1* [b] |
| | No | 44 (88) | 175 (78) | 48 (75) | | | |
| Electronic device before falling asleep (/ Week) | None | 0 (0) | 0 (0) | 1 (2) | *0.72* [b] | *0.11* [b] | *0.95* [b] |
| | 1 - 3 | 2 (4) | 6 (3) | 4 (6) | | | |
| | 4 - 6 | 3 (6) | 19 (8) | 3 (5) | | | |
| | 7 | 45 (90) | 200 (89) | 56 (87) | | | |
| Sleep concerns related to sports | Never | 25 (50) | 72 (32) | 13 (20) | *0.1* [b] | *0.2* [b] | *0.003* [b] |
| | Rarely | 14 (28) | 65 (29) | 18 (28) | | | |
| | Sometimes | 9 (18) | 60 (27) | 20 (31) | | | |
| | Frequently | 2 (4) | 24 (10) | 10 (16) | | | |
| | Always | 0 (0) | 4 (2) | 3 (5) | | | |
| Sleep concerns not related to sports | Never | 20 (40) | 41 (18) | 4 (6) | *0.002* [b] | *0.0005* [b] | *< $10^{-4}$* [b] |
| | Rarely | 12 (24) | 43 (19) | 4 (6) | | | |
| | Sometimes | 12 (24) | 65 (29) | 21 (32) | | | |
| | Frequently | 4 (8) | 60 (27) | 22 (34) | | | |
| | Always | 2 (4) | 16 (7) | 13 (20) | | | |

*Statistical analysis: median [IQR], number (percentage), a. 2-tailed Student test (t-Test) for unpaired data, b. Chi-square test of independency (two-tailed), c. 2-tailed Fisher's exact test for categorical data.*

**Table S4.** (suite) Univariate analyses of sleep disrupters between three different groups according to PSQI.

| **Variables** | **Modality** | **PSQI [0- 5] (Group1) N = 50** | **PSQI [6- 11] (Group2) N = 225** | **PSQI [12-21] (Group3) N = 64** | ***p Group 1*2*** | ***p Group 2*3*** | ***p Group 1*3*** |
|---|---|---|---|---|---|---|---|
| Naps (/ Week) | None | 29 (58) | 124 (55) | 34 (53) | *1* [b] | *0.2* [b] | *0.3*[b] |
| | 1 - 2 | 17 (34) | 78 (35) | 17 (26) | | | |
| | 3 - 4 | 3 (6) | 15 (7) | 8 (13) | | | |
| | 5 - 7 | 1 (2) | 8 (4) | 5 (8) | | | |
| Do you get up every morning at the same time? | Never | 3 (6) | 10 (4) | 6 (9) | *0.4* [b] | *0.3* [b] | *0.8* [b] |
| | Rarely | 6 (12) | 45 (20) | 11 (17) | | | |
| | Sometimes | 12 (24) | 67 (30) | 15 (23) | | | |
| | Frequently | 27 (54) | 97 (43) | 28 (44) | | | |
| | Always | 2 (4) | 6 (3) | 4 (7) | | | |
| Do you go to bed every night at the same time? | Never | 5 (10) | 19 (8) | 13 (20) | *0.2* [b] | *0.03* [b] | *0.1* [b] |
| | Rarely | 8 (16) | 48 (21) | 18 (28) | | | |
| | Sometimes | 12 (24) | 80 (36) | 14 (22) | | | |
| | Frequently | 24 (48) | 76 (34) | 19 (30) | | | |
| | Always | 1 (2) | 2 (1) | 0 (0) | | | |

*Statistical analysis: median [IQR], number (percentage), a. 2-tailed Student test (t-Test) for unpaired data, b. Chi-square test of independency (two-tailed), c. 2-tailed Fisher's exact test for categorical data.*
